# A Stress Process Model of Postpartum Depressive Symptoms Among Thai Mothers: A Structural Equation Modeling Approach

**DOI:** 10.3390/nursrep16080269

**Published:** 2026-08-03

**Authors:** Apinya Chaiwongsa, Nantaporn Sansiriphun, Patraporn Bhatarasakoon, Nonglak Chaloumsuk

**Affiliations:** Faculty of Nursing, Chiang Mai University, 110/406 Inthawaroros Road, Suthep, Mueang Chiang Mai, Chiang Mai 50200, Thailand; apinya_chaiwongsa@cmu.ac.th (A.C.); nantaporn.san@cmu.ac.th (N.S.); patraporn.t@cmu.ac.th (P.B.)

**Keywords:** postpartum depressive symptoms, Stress Process Model, structural equation modeling, social support, postpartum women, nursing

## Abstract

**Background/Objectives**: Postpartum depressive symptoms are a major maternal mental health concern associated with adverse outcomes for mothers and infants. Although biological, psychological, and social factors have been associated with postpartum depressive symptoms, limited evidence has examined their interrelationships within a theoretically grounded framework among Thai mothers. This study aimed to test a hypothesized Stress Process Model of factors associated with postpartum depressive symptoms among Thai mothers using structural equation modeling (SEM). **Methods**: A cross-sectional study was conducted among 462 Thai mothers attending routine 4–6-week postnatal follow-up clinics at three tertiary hospitals in Northern Thailand. Participants were recruited using proportional allocation and consecutive sampling. Data were collected between April and June 2025 using standardized measures of postpartum depressive symptoms and standardized measures of self-perceived burden, parenting stress, social support, self-esteem, mastery, and coping, together with demographic and clinical data. Data were analyzed using descriptive statistics and SEM. **Results**: Using the prespecified Edinburgh Postnatal Depression Scale cutoff score of ≥11, 30.95% of participants screened positive for probable postpartum depressive symptoms. The final structural model demonstrated acceptable fit to the data (χ^2^/df = 2.95, RMSEA = 0.07, CFI = 0.94, TLI = 0.92, and SRMR = 0.05) and explained 65.10% of the variance in postpartum depressive symptoms. Self-perceived burden and parenting stress showed significant positive direct associations with postpartum depressive symptoms, whereas social support showed significant inverse direct and indirect associations. Perinatal complications were directly associated with postpartum depressive symptoms and indirectly associated through self-perceived burden and parenting stress. Mastery also showed a significant inverse indirect association. **Conclusions**: The findings support theoretically specified associations consistent with the Stress Process Model. Because all variables were assessed concurrently, the identified pathways should be interpreted as statistical associations rather than evidence of temporal ordering or causal mechanisms.

## 1. Introduction

Postpartum depressive symptoms are a major maternal mental health concern with adverse consequences for mothers, infants, and families. Although many women adapt successfully to motherhood, a substantial proportion experience depressive symptoms during the early postpartum period, including during the first 4–6 weeks after childbirth [1,2]. Globally, the prevalence of postpartum depressive symptoms has been estimated at 17.22% [3], while a systematic review in ASEAN countries reported a pooled prevalence of 25.24%, with Thailand showing the highest estimated prevalence in the region at 36.17% [4]. These findings indicate that postpartum depressive symptoms remain a significant and persistent maternal health problem in Thailand.

Postpartum depression has wide-ranging effects on maternal functioning and infant development. Women with postpartum depressive symptoms may experience anxiety, sleep disturbance, fatigue, impaired daily functioning, reduced quality of life, relationship difficulties, and increased risk of suicidal ideation [5,6,7]. Postpartum depressive symptoms are also associated with impaired maternal role functioning, shorter breastfeeding duration, lower breastfeeding self-efficacy, and difficulties adapting to motherhood [7,8,9]. Beyond maternal health, postpartum depressive symptoms may disrupt mother–infant bonding and attachment and have been linked to poorer infant physical health, sleep, motor development, and cognitive and language outcomes [7,10,11,12]. Persistent maternal depressive symptoms may also have long-term effects, including children’s internalizing problems at school age [13].

A substantial body of research has identified factors associated with postpartum depressive symptoms across biological, psychological, social, and obstetric domains. Biological studies have emphasized neuroendocrine, inflammatory, and genetic mechanisms underlying vulnerability to postpartum depressive symptoms [14], while psychosocial research has highlighted stress exposure, interpersonal difficulties, social adversity, and maternal role adjustment [15,16,17,18]. More recent studies have proposed multidimensional approaches that integrate biological, psychological, social, and obstetric factors to better explain postpartum depressive symptoms risk [19,20,21,22]. However, many studies continue to examine isolated risk factors or direct associations, providing limited insight into how stressors and psychosocial resources may operate together through interconnected pathways.

Established determinants of postpartum depressive symptoms include prior or antenatal mental health problems, unintended pregnancy, marital or partner relationship difficulties, difficult infant temperament, and socioeconomic adversity. The present study, however, was not designed to construct an exhaustive etiological or predictive model incorporating all established determinants. Instead, a focused and parsimonious structural model was specified to examine stress-process mechanisms relevant to early postpartum clinical stress, maternal role-related strain, and psychosocial resources. This approach enabled the simultaneous examination of an objectively documented clinical stressor, secondary subjective stressors, and potentially modifiable personal and social resources while preserving theoretical coherence and ensuring that all constructs were adequately operationalized.

The Stress Process Model (SPM), proposed by Pearlin et al. [23,24], provides a framework for understanding the theoretically specified associations among primary and secondary stressors, stress proliferation, psychosocial resources, and mental health outcomes. The model is particularly relevant to the postpartum period, during which mothers may concurrently experience physical recovery, infant-care demands, maternal role transition, and changes in family relationships. The study-specific application of the SPM, including the conceptualization of the study variables and the rationale for the hypothesized direct and indirect associations, is described in detail in Section 2.8.

The Thai sociocultural context further supports the need for a theory-driven examination of postpartum depressive symptoms. Traditional expectations of motherhood in Thailand emphasize maternal responsibility, family harmony, and women’s roles in caregiving and domestic functioning. Postpartum mothers who experience physical limitations, perinatal complications, or difficulty fulfilling expected maternal roles may therefore experience heightened perceived burden and psychological distress. At the same time, support from husbands, extended family members, and community networks may reduce caregiving demands and provide emotional reassurance. Despite recognition of these cultural and psychosocial factors, the pathways through which stressors and resources interact to influence postpartum depressive symptoms among Thai mothers remain insufficiently examined. Most studies in Thailand have focused on identifying individual associated factors of postpartum depressive symptoms using correlational or regression-based analyses.

Therefore, this study aimed to test a Stress Process Model of postpartum depressive symptoms among Thai mothers using structural equation modeling. Specifically, the study examined the direct and indirect associations of perinatal complications, self-perceived burden, parenting stress, self-esteem, mastery, coping, and social support with postpartum depressive symptoms. By testing these pathways within a theoretically grounded model, this study seeks to provide a more comprehensive understanding of postpartum depressive symptoms in the Thai context and to inform nursing and midwifery interventions that address both clinical stressors and psychosocial resources.

## 2. Literature Review and Hypothesis

Guided by the Stress Process Model, the following subsections summarize the key theoretical and empirical evidence supporting the hypothesized associations.

### 2.1. Perinatal Complications and Postpartum Depressive Symptoms

Perinatal complications encompass a broad range of maternal conditions that may occur during pregnancy, childbirth, or the immediate postpartum period [25]. Perinatal complications occurring during pregnancy, childbirth, or the immediate postpartum period have been consistently associated with greater postpartum depressive symptoms [26,27,28,29,30,31,32,33,34]. Evidence also suggests a cumulative pattern, whereby depressive symptoms increase with the number of complications experienced [27,34]. Within the Stress Process Model, perinatal complications were conceptualized as primary stressors that may be directly associated with postpartum depressive symptoms and indirectly associated through self-perceived burden and parenting stress, which were conceptualized as secondary stressors [23,24,35,36].

**H1.**  *Perinatal complications have a positive direct association with postpartum depressive symptoms and positive indirect associations through self-perceived burden and parenting stress among Thai mothers.*

### 2.2. Self-Perceived Burden and Postpartum Depressive Symptoms

Self-perceived burden (SPB) refers to feelings of guilt, distress, and diminished self-worth arising from the perception that one’s illness, limitations, or care needs negatively affect others [37]. During the postpartum period, physical limitations, perinatal complications, and difficulties fulfilling expected maternal or family roles may contribute to these perceptions. Previous studies have demonstrated positive associations between SPB and depressive symptoms, including among women hospitalized with threatened preterm labor [38]. Equity theory further suggests that psychological distress may occur when individuals perceive an imbalance between the support they receive and their ability to reciprocate within important relationships [37]. Within the Stress Process Model, SPB was conceptualized as a secondary stressor positively associated with postpartum depressive symptoms.

**H2.**  *Self-perceived burden has a positive direct association with postpartum depressive symptoms among Thai mothers.*

### 2.3. Parenting Stress and Postpartum Depressive Symptoms

Parenting stress refers to stress associated with the demands of caring for and raising children [39]. During the transition to motherhood, increased caregiving responsibilities, sleep disruption, emotional adjustment, and role strain may contribute to heightened parenting stress. Previous studies have consistently demonstrated positive associations between parenting stress and postpartum depressive symptoms [40,41], as well as poorer maternal psychological well-being [42]. Within the Stress Process Model, parenting stress was conceptualized as a secondary stressor directly associated with postpartum depressive symptoms.

**H3.**  *Parenting stress has a positive direct association with postpartum depressive symptoms among Thai mothers.*

### 2.4. Social Support and Postpartum Depressive Symptoms

Social support refers to the perceived availability of emotional, informational, and practical assistance from family members, friends, and significant others [43]. Previous evidence has consistently demonstrated inverse associations between social support and postpartum depressive symptoms. A systematic review and meta-analysis found that women with limited social support were more likely to experience postpartum depressive symptoms than those with adequate support [44]. Similarly, mothers reporting moderate or low social support had higher odds of postpartum depressive symptoms during the first year after childbirth [45]. Within the Stress Process Model, social support was conceptualized as an interpersonal psychosocial resource inversely associated with postpartum depressive symptoms.

**H4.**  *Social support has a negative direct association with postpartum depressive symptoms among Thai mothers.*

### 2.5. Self-Esteem and Postpartum Depressive Symptoms

Self-esteem refers to an individual’s overall evaluation of self-worth and self-acceptance [46]. Previous studies across different cultural contexts have demonstrated inverse associations between self-esteem and postpartum depressive symptoms [47,48]. Studies among Thai mothers have similarly reported that higher self-esteem is associated with lower levels of postpartum depressive symptoms [41]. Within the Stress Process Model, self-esteem was conceptualized as an intrapersonal psychosocial resource inversely associated with postpartum depressive symptoms.

**H5.**  *Self-esteem has a negative direct association with postpartum depressive symptoms among Thai mothers.*

### 2.6. Mastery and Postpartum Depressive Symptoms

Mastery refers to an individual’s perception of control over life circumstances and the ability to manage environmental challenges [49]. Previous evidence has demonstrated inverse associations between mastery and depressive symptoms and suggests that mastery may be relevant to responses to prenatal and postpartum stressors [50]. Within the Stress Process Model, mastery was conceptualized as an intrapersonal psychosocial resource associated with lower levels of postpartum depressive symptoms, including through its hypothesized associations with secondary stressors.

**H6.**  *Mastery has a negative direct association with postpartum depressive symptoms among Thai mothers.*

### 2.7. Coping and Postpartum Depressive Symptoms

Coping refers to cognitive and behavioral efforts used to manage stressful situations [49]. Previous studies have indicated that coping strategies may be differentially associated with postpartum depressive symptoms. Problem-focused coping has been associated with lower levels of postpartum depressive symptoms, whereas avoidant and emotion-focused coping have been associated with greater symptom levels [51,52]. Within the Stress Process Model, coping was conceptualized as a behavioral psychosocial resource associated with postpartum depressive symptoms [16].

**H7.**  *Coping has a negative direct association with postpartum depressive symptoms among Thai mothers.*

### 2.8. Theoretical Framework

The Stress Process Model (SPM), proposed by Pearlin et al. [23,24], provided the conceptual foundation for this study. The SPM explains mental health outcomes through the interrelationships among primary stressors, secondary stressors, psychosocial resources, and stress outcomes. In the present study, perinatal complications were conceptualized as primary stressors, whereas self-perceived burden and parenting stress were conceptualized as secondary stressors reflecting maternal role demands and caregiving strain. Social support, mastery, self-esteem, and coping were conceptualized as complementary psychosocial resources or responses relevant to mothers’ adaptation to postpartum challenges. Postpartum depressive symptoms were conceptualized as the mental health outcome.

The selection and theoretical ordering of these variables were based on their distinct roles within the stress process. Perinatal complications were selected as primary stressors because they represent objectively documented health-related events that may be associated with physical recovery, maternal expectations, and maternal role functioning. Self-perceived burden and parenting stress were positioned as secondary stressors because they represent subjective role-related and caregiving strains theoretically associated with primary stressors within the stress proliferation process.

Social support, mastery, self-esteem, and coping were included because they represent complementary interpersonal, intrapersonal, and behavioral dimensions of the stress process. Social support represents an interpersonal resource available to mothers; mastery reflects perceived control over life circumstances; self-esteem reflects global self-appraisal; and coping represents cognitive and behavioral responses to stressful demands. These variables were therefore examined in relation to postpartum depressive symptoms and, where specified in the hypothesized model, secondary stressors.

Based on the SPM and previous empirical evidence, the proposed model specified direct associations of perinatal complications, self-perceived burden, parenting stress, social support, mastery, self-esteem, and coping with postpartum depressive symptoms. It also specified indirect associations of perinatal complications with postpartum depressive symptoms through self-perceived burden and parenting stress. These indirect pathways reflect the theoretical concept of stress proliferation, in which primary health-related stressors are theorized to be associated with maternal role-related and caregiving strains, which are, in turn, associated with postpartum depressive symptoms. Pathways involving psychosocial resources represent their hypothesized associations with secondary stressors and/or postpartum depressive symptoms, as specified in the structural model. Structural equation modeling was therefore used to examine the simultaneous direct and indirect associations among primary stressors, secondary stressors, psychosocial resources, and postpartum depressive symptoms among Thai mothers.

Because the psychosocial variables and postpartum depressive symptoms were assessed concurrently at 4–6 weeks postpartum, the directions of the specified pathways represent theoretical ordering based on the SPM rather than established temporal or causal relationships. Accordingly, the terms direct, indirect, and total associations refer to the statistical decomposition of relationships estimated within the structural equation model and should not be interpreted as evidence of causal mediation.

## 3. Materials and Methods

### 3.1. Aim and Objectives

This study aimed to test a theoretically grounded Stress Process Model of factors associated with postpartum depressive symptoms among Thai mothers. Specifically, the study examined the hypothesized direct and indirect associations among perinatal complications, secondary psychosocial stressors, psychosocial resources, and postpartum depressive symptoms.

The study was guided by the following objectives:

1. To examine the direct associations of perinatal complications, self-perceived burden, parenting stress, social support, self-esteem, mastery, and coping with postpartum depressive symptoms among Thai mothers.
2. To examine the hypothesized indirect associations of perinatal complications and psychosocial resources with postpartum depressive symptoms through self-perceived burden and parenting stress, as specified in the model.
3. To evaluate the overall fit and explanatory power of the hypothesized Stress Process Model of postpartum depressive symptoms among Thai mothers using structural equation modeling.

### 3.2. Design

This study employed a cross-sectional analytical design to test a theoretically derived model of postpartum depressive symptoms among Thai mothers. Structural equation modeling was used to examine the direct and indirect relationships among stressors, psychosocial resources, and postpartum depressive symptoms. This manuscript was prepared in accordance with the Strengthening the Reporting of Observational Studies in Epidemiology (STROBE) guidelines for observational studies.

### 3.3. Setting

Data were collected from routine 4–6-week postnatal follow-up clinics at three purposively selected tertiary hospitals under the Ministry of Public Health in Northern Thailand: Maharaj Nakorn Chiang Mai Hospital, Lamphun Hospital, and Lampang Hospital. These hospitals serve as major regional referral centers and provide advanced maternity and neonatal care, including the management of complex obstetric and perinatal conditions. The selected settings therefore provided access to a clinically diverse population of postpartum mothers with varying levels of perinatal and neonatal health complexity.

### 3.4. Participants and Sampling

The accessible population consisted of Thai postpartum women attending routine 4–6-week postnatal follow-up clinics at the three selected tertiary hospitals in Northern Thailand. A multi-stage sampling approach was used. In the first stage, one geographical region of Thailand was selected by simple random sampling, resulting in the selection of the northern region. In the second stage, three tertiary hospitals in this region were purposively selected based on their roles as referral centers for maternity and neonatal care and their capacity to provide access to a clinically diverse postpartum population. In the third stage, proportional allocation was used to determine the re-quired number of participants from each hospital based on annual postnatal clinic service volumes. Eligible participants were then recruited consecutively at each site until the allocated sample size was reached.

The accessible population across the three selected hospitals comprised 3416 Thai postpartum women receiving postnatal follow-up care. The corresponding accessible populations were 1366 women at Maharaj Nakorn Chiang Mai Hospital, 1107 at Lampang Hospital, and 943 at Lamphun Hospital. Based on proportional allocation, the final sample of 462 participants was distributed across the study sites as follows: 185 participants from Maharaj Nakorn Chiang Mai Hospital, 150 from Lampang Hospital, and 127 from Lamphun Hospital. This approach enabled the study to include postpartum women from different tertiary care settings with varying levels of obstetric and neonatal health complexity.

Registered nurse-midwives screened potential participants for eligibility by reviewing postnatal clinic appointment lists and relevant medical records and by assessing whether women had any acute clinical conditions requiring immediate care before enrollment. Women were included if they were Thai, aged 20 years or older, 4–6 weeks postpartum, had given birth to a living infant, were able to read and communicate in Thai, and were willing to participate and provide written informed consent. Women were excluded if they presented with acute medical conditions requiring immediate clinical attention, such as high-grade fever, active vaginal bleeding, or uncontrolled hypertension; or had a documented history of clinical depression or psychiatric treatment before the current pregnancy.

The sample size was determined to support the proposed structural equation model. Following the guideline of 5–10 participants per estimated parameter [53], and given that the hypothesized model included 84 free parameters comprising 21 intercepts, 21 measurement-error variances, one factor variance, seven residual variances, 13 factor loadings, and 21 regression coefficients, a minimum sample of 420 participants was required using a 5:1 ratio. An a priori SEM sample size estimation using Soper’s SEM calculator, based on eight latent variables, 21 observed variables, an anticipated effect size of 0.20, and statistical power of 0.80, also supported this requirement. To account for potential non-response or incomplete data, the sample size was increased by 10%, resulting in a final target sample of 462 participants. This sample size was considered adequate for stable maximum likelihood estimation in SEM [54].

### 3.5. Instruments

Data were collected using a questionnaire booklet comprising eight instruments, including a researcher-developed data recording form for sociodemographic and obstetric characteristics. Three instruments were translated into Thai following the World Health Organization (WHO) back-translation protocol [55], and four previously validated Thai instruments were used with permission.

#### 3.5.1. Demographic Data Recording Form

This form collected personal characteristics, including age, marital status, education, occupation, and income, as well as obstetric characteristics, including gravida, parity, and mode of delivery. The form also included a researcher-developed 12-item clinical checklist to assess documented perinatal complications across three phases: pregnancy, childbirth, and the immediate postpartum period. Pregnancy-related complications comprised hypertensive disorders of pregnancy, gestational diabetes mellitus, anemia, infections, placenta previa, and antenatal depression. Childbirth-related complications comprised preterm labor, fetal distress, instrumental delivery, and emergency cesarean section. Postpartum complications comprised postpartum hemorrhage and postpartum anemia. Each condition was coded as absent (0) or present (1), and the item scores were summed to produce a total score ranging from 0 to 12. Higher scores indicated exposure to a greater number of documented perinatal complications. The measure was designed as an unweighted cumulative exposure index rather than an index of clinical severity. Equal weighting was used because no validated severity-weighting scheme applicable across these heterogeneous conditions had been prespecified in the study protocol. Accordingly, the score reflects the number of documented complications and does not account for differences in severity, duration, treatment intensity, or psychological impact.

#### 3.5.2. Self-Perceived Burden Scale (SPBS)

The 10-item SPBS assesses a mother’s perception of being a physical, emotional, or economic burden on others [56]. Items are rated on a 5-point Likert scale ranging from 1 (never) to 5 (always). Total scores range from 10 to 50, with higher scores indicating greater perceived burden. The original scale reported a Content Validity Index (CVI) of 0.92. In the present study, the Thai version demonstrated strong internal consistency reliability (Cronbach’s alpha = 0.93).

#### 3.5.3. Parental Stress Scale (PSS)

The 18-item PSS assesses parenting-related strain across four domains: parental rewards, stressors, lack of control, and satisfaction [39]. Items are rated on a 5-point Likert scale. After reverse-coding positive items, total scores range from 18 to 90, with higher scores indicating greater parenting stress. In the present study, the Thai version demonstrated strong internal consistency reliability (Cronbach’s alpha = 0.89).

#### 3.5.4. Multidimensional Scale of Perceived Social Support (r-Thai MSPSS)

The 12-item revised Thai version of the r-Thai MSPSS assesses perceived support from family, friends, and significant others [57]. Items are rated on a 7-point Likert scale ranging from 1 (very strongly disagree) to 7 (very strongly agree). Total scores range from 12 to 84, with higher scores indicating greater perceived social support. In the present study, the instrument demonstrated strong internal consistency reliability (Cronbach’s alpha = 0.92).

#### 3.5.5. Rosenberg Self-Esteem Scale (RSES)

The 10-item Thai version of the RSES assesses global self-worth and self-acceptance using a 4-point Likert scale [58]. Total scores range from 10 to 40, with higher scores indicating higher self-esteem. In the present study, the scale demonstrated strong internal consistency reliability (Cronbach’s alpha = 0.89).

#### 3.5.6. Personal Mastery Scale

The 7-item Personal Mastery Scale assesses perceived control over life circumstances [49]. Items are rated on a 4-point Likert scale, with total scores ranging from 7 to 28. Higher scores indicate greater perceived mastery. The scale was translated into Thai following the WHO back-translation protocol. In the present study, the Thai version demonstrated acceptable internal consistency reliability (Cronbach’s alpha = 0.82).

#### 3.5.7. Brief Coping Orientation for Problem Experiences (Brief COPE)

The 28-item Thai version of the Brief COPE assesses 14 coping subscales, which can be grouped into problem-focused coping, active emotional coping, and avoidant emotional coping dimensions [59]. Items are rated on a 4-point Likert scale. Higher scores indicate greater use of coping strategies. In the present study, the instrument demonstrated acceptable internal consistency reliability (Cronbach’s alpha = 0.84).

#### 3.5.8. Edinburgh Postnatal Depression Scale (EPDS)

The 10-item Thai version of the EPDS assesses depressive symptoms experienced during the previous seven days [60]. Total scores range from 0 to 30, with higher scores indicating greater symptom severity. For descriptive and prevalence analyses, a score of ≥11 was prespecified as the analytic screening cutoff indicating a positive screen for probable postpartum depressive symptoms. At this cutoff, the original Thai development study reported 100% sensitivity and 88% specificity [61]. This analytic cutoff was distinct from the higher score of ≥13 used solely to initiate clinical referral under institutional safety protocols; the referral threshold did not affect the classification of the study outcome or the structural equation modeling analysis. The EPDS is a screening instrument and does not establish a clinical diagnosis. In the structural equation model, the EPDS score was analyzed as a measure of postpartum depressive symptoms. In the present study, the EPDS demonstrated strong internal consistency reliability (Cronbach’s alpha = 0.90).

### 3.6. Data Collection

Following ethical approval and institutional permission, formal requests were submitted to the administrative directors of each participating hospital. Briefing sessions were conducted with nursing department heads and research assistants to ensure consistency in participant screening, recruitment, and data collection procedures across the three study sites.

Qualified research assistants initially screened postnatal clinic appointment lists to identify potentially eligible participants. Eligible mothers attending routine 4–6-week postnatal follow-up visits were consecutively approached in a private counseling or health education room. The researchers explained the study purpose, procedures, voluntary nature of participation, confidentiality, and the right to withdraw at any time without consequences. Written informed consent was obtained from all participants before data collection.

Participants completed the questionnaire booklet independently, which required approximately 45–60 min. They could complete the questionnaires at the clinic or return them within two weeks using a provided sealed return envelope. All returned questionnaires were checked for completeness.

### 3.7. Data Analysis

Data screening and descriptive analyses were conducted using SPSS version 21.0, while structural equation modeling was performed using Mplus, version 8 Sociodemographic and obstetric characteristics were summarized using frequencies, percentages, means, and standard deviations. Pearson’s correlation coefficients were used to examine bivariate relationships among continuous variables.

For descriptive analyses, participants with EPDS scores ≥ 11 were classified as screening positive for probable postpartum depressive symptoms, and the corresponding prevalence was calculated. For Pearson’s correlation and structural equation modeling analyses, the total EPDS score was treated as a continuous variable.

Before testing the structural model, assumptions for SEM were assessed. The dataset was complete (*N* = 462), with no missing values. Multivariate outliers were examined using Mahalanobis distance, and no cases were excluded. Univariate normality was supported, with skewness values ranging from −0.15 to 0.81 and kurtosis values ranging from −1.22 to −0.059, which were within acceptable thresholds. These results supported the use of maximum likelihood estimation. Linearity and homoscedasticity were assessed through residual scatterplots. Multicollinearity was also examined, with bivariate correlations ranging from −0.28 to 0.657 and variance inflation factor values ranging from 1.23 to 2.34, indicating no evidence of multicollinearity.

The measurement and structural components of the model were evaluated using structural equation modeling. For the measurement model, standardized factor loadings, composite reliability (CR), average variance extracted (AVE), and Cronbach’s alpha were examined collectively to assess the measurement properties of each latent construct. CR was used to assess construct reliability, whereas AVE was used to assess convergent validity. CR values of 0.70 or higher and AVE values of 0.50 or higher were considered indicative of acceptable construct reliability and convergent validity, respectively. However, these indices were interpreted together with the standardized factor loadings and Cronbach’s alpha rather than relying on any single criterion.

Overall model fit was assessed using multiple indices, including the chi-square/degrees of freedom ratio (χ^2^/df < 3), root mean square error of approximation (RMSEA < 0.08), comparative fit index (CFI > 0.90), Tucker–Lewis index (TLI > 0.90), and standardized root mean square residual (SRMR < 0.08) [62]. Standardized path coefficients, indirect associations, and coefficients of determination (R^2^) were examined to evaluate the magnitude and direction of the modeled relationships and the proportion of variance explained in the outcome.

The initial theoretically specified model was evaluated before any respecification. When the initial model did not demonstrate adequate fit, modification indices, fully standardized expected parameter changes, and theoretical interpretability were used to identify potential areas of local model misfit. User-specified respecifications were introduced sequentially, and the model was re-estimated after each step. No structural regression or measurement specification was added or removed during this process.

### 3.8. Ethical Considerations

This study was approved by the Research Ethics Committee of the Faculty of Nursing, Chiang Mai University (Study Code: 2568-EXP007) and the Institutional Review Boards of all participating hospitals. All procedures were conducted in accordance with the ethical principles of the Declaration of Helsinki. Participants were informed about the study purpose, procedures, voluntary nature of participation, confidentiality, and their right to withdraw at any time without affecting their standard medical care. Written informed consent was obtained from all participants prior to data collection. To protect confidentiality, all data were anonymized using numerical codes, stored on a password-protected computer, and accessible only to the primary research team. Participants with EPDS scores ≥ 13 were informed of their screening results confidentially and referred for further clinical assessment and supportive care in accordance with the participating institutions’ safety protocols. This clinical action threshold was distinct from the analytic screening cutoff of ≥11 used to estimate the prevalence of a positive screen for probable postpartum depressive symptoms and was not used to classify the study outcome.

## 4. Results

### 4.1. Characteristics of Participants

A total of 462 Thai postpartum women participated in this study. Their sociodemographic and obstetric characteristics are presented in Table 1. The mean age of participants was 30.98 years (SD = 5.07; range = 20–47), and most were aged 30–39 years. The majority were married and had completed at least high school or vocational education. Most participants were salaried employees, and more than half reported a monthly family income of 10,001–20,000 Thai baht. Regarding family context, more than half lived in extended family households, whereas approximately two-fifths lived in nuclear families.

In terms of obstetric characteristics, slightly more than half of the participants were multigravida, and most had one or two living children. Most participants delivered at term, and vaginal delivery was the most common mode of delivery. Neonatal complications were reported in a minority of cases, and most newborns were hospitalized for no more than five days. Most participants had no history of hospital admission during pregnancy. These characteristics provide contextual information for interpreting the stress process model, particularly in relation to maternal role demands, family support, and clinical complexity during the early postpartum period.

### 4.2. Postpartum Depression Among Participants

Using the prespecified EPDS cutoff of ≥11, 143 participants (30.95%) screened positive for probable postpartum depressive symptoms, whereas 319 participants (69.05%) scored below the cutoff. EPDS scores ranged from 0 to 18, with a mean of 7.74 (SD = 4.72). These classifications represent screening status and do not constitute clinical diagnoses, as shown in Table 2.

### 4.3. Measurement Model Assessment

Before testing the structural model, confirmatory factor analysis was conducted to evaluate the measurement properties of the six latent constructs: self-perceived burden, parenting stress, social support, self-esteem, mastery, and coping. Perinatal complications were treated as an observed count variable and were therefore not included in the measurement model. The EPDS total score was specified as the observed measure of postpartum depressive symptoms in the structural model.

As shown in Table 3, all standardized factor loadings were statistically significant. Self-perceived burden, parenting stress, social support, and self-esteem demonstrated adequate measurement properties, with standardized factor loadings ranging from 0.692 to 0.946, composite reliability values ranging from 0.819 to 0.913, and average variance extracted values ranging from 0.599 to 0.780. These four constructs met the recommended thresholds for construct reliability and convergent validity.

Mastery and coping showed weaker measurement properties. For mastery, standardized factor loadings ranged from 0.556 to 0.683, with a CR of 0.556 and an AVE of 0.388. For coping, standardized factor loadings ranged from 0.197 to 0.751, with a CR of 0.477 and an AVE of 0.272. Thus, neither construct met the recommended thresholds for construct reliability or convergent validity. The indicators were retained to preserve the prespecified theoretical structure of the model; however, findings involving mastery and coping should be interpreted cautiously.

### 4.4. Correlations Among Study Variables

Pearson’s correlation analysis was conducted to examine the bivariate relationships among the study variables before structural model testing. As shown in Table 4, postpartum depressive symptoms were positively correlated with perinatal complications, self-perceived burden, and parenting stress, and negatively correlated with social support, self-esteem, mastery, and coping. The strongest association was observed between perinatal complications and postpartum depressive symptoms.

Intercorrelations among the study variables were generally in the expected directions and consistent with the Stress Process Model. Perinatal complications, self-perceived burden, and parenting stress were positively correlated with one another, whereas psychosocial resources, including social support, self-esteem, mastery, and coping, were generally negatively correlated with stress-related variables and positively correlated with one another. The magnitude of correlations among predictor variables did not indicate severe multicollinearity, supporting their inclusion in the subsequent structural equation model.

### 4.5. Structural Model Development and Final Model

The hypothesized structural model was tested using structural equation modeling with maximum likelihood estimation. The initial model did not meet all prespecified fit criteria, χ^2^ = 592.368, df = 149, *p* < 0.001, χ^2^/df = 3.98, RMSEA = 0.080, 90% CI [0.074, 0.087], SRMR = 0.085, CFI = 0.904, and TLI = 0.877. Model respecification was guided by modification indices, fully standardized expected parameter changes (StdYX EPCs), and theoretical interpretability.

Five user-specified respecifications were introduced sequentially, with the model re-estimated after each step. Residual covariances were specified for CO3–CO2, PS4–PS3, and PS2–PS1, representing localized associations not fully explained by their respective latent constructs. A covariance between perinatal complications (C2) and social support (SS) was then specified, incorporating C2 into the modeled covariance structure and resulting in additional C2-related parameter estimation. Finally, a covariance was specified between the disturbances of parenting stress (PS) and self-perceived burden (SPB), reflecting residual covariation beyond their specified predictors. No structural regression or measurement specifications were changed.

**Figure 1:**
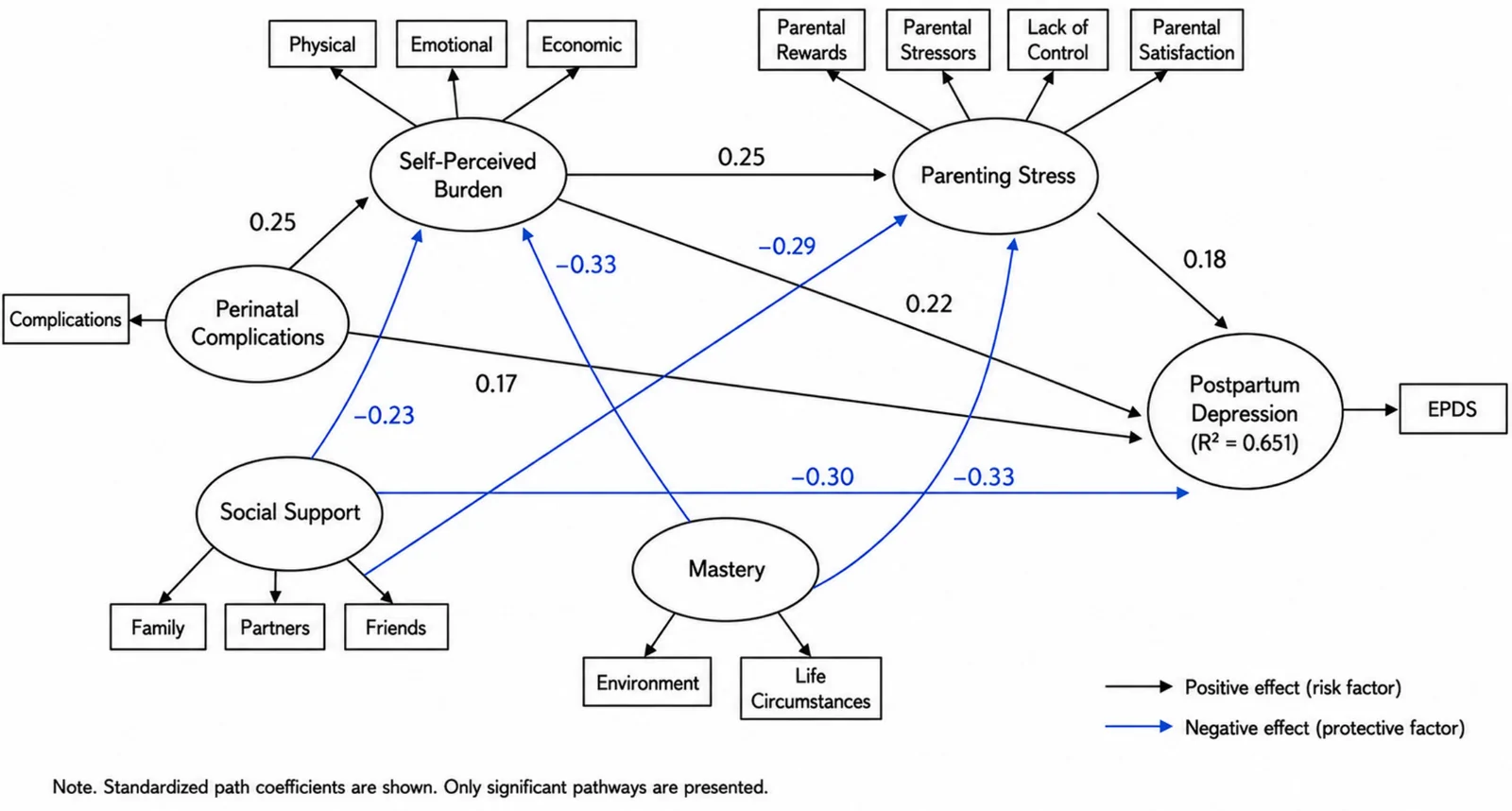
Final structural model of postpartum depressive symptoms among Thai mothers.

The final model demonstrated acceptable fit, χ^2^ = 419.363, df = 142, *p* < 0.001, χ^2^/df = 2.95, RMSEA = 0.065, 90% CI [0.058, 0.072], SRMR = 0.046, CFI = 0.940, and TLI = 0.919, and explained 65.10% of the variance in postpartum depressive symptoms. The sequential respecifications and corresponding statistical evidence and fit indices are presented in Table 5; the final model is shown in Figure 1.

### 4.6. Direct, Indirect, and Total Associations

In this cross-sectional structural equation model, the direct, indirect, and total estimates represent the statistical decomposition of modeled associations rather than causal effects. Table 6 presents the standardized direct, indirect, and total associations of the study variables with postpartum depressive symptoms. Perinatal complications showed significant positive direct and indirect associations with postpartum depressive symptoms, resulting in a significant positive total association. Self-perceived burden and parenting stress also showed significant positive direct associations with postpartum depressive symptoms.

Among the psychosocial resources, social support demonstrated the strongest inverse association, with significant negative direct, indirect, and total associations with postpartum depressive symptoms. Mastery did not demonstrate a significant direct association; however, it showed a significant negative indirect association and a significant negative total association. In contrast, self-esteem and coping did not demonstrate statistically significant direct, indirect, or total associations with postpartum depressive symptoms in the final model.

## 5. Discussion

This study tested a theoretically grounded Stress Process Model of postpartum depressive symptoms among Thai mothers using structural equation modeling. The final model demonstrated acceptable fit and explained 65.10% of the variance in postpartum depressive symptoms, suggesting that the Stress Process Model provides a useful framework for understanding postpartum depressive symptoms during the early postpartum period. Overall, the findings suggest that postpartum depressive symptoms are associated with a cascading stress process in which primary clinical stressors, secondary psychological stressors, and psychosocial resources operate through interconnected direct and indirect pathways.

Within the proposed model, perinatal complications functioned as primary stressors and were associated with postpartum depressive symptoms through both direct and indirect pathways. These findings are consistent with the Stress Process Model, which proposes that health-related stressors may initiate subsequent stress processes that influence mental health outcomes. Perinatal complications may increase emotional distress through physical exhaustion, uncertainty regarding maternal and infant health, and disruption of expectations surrounding pregnancy, childbirth, and postpartum recovery. Their indirect associations through self-perceived burden and parenting stress further support the concept of stress proliferation, whereby initial stressors contribute to the development of secondary stressors that more proximally influence psychological well-being [23,24]. This finding is consistent with previous studies reporting associations between obstetric complications and increased risk of postpartum depressive symptoms [63].

At the intermediate level of the model, self-perceived burden and parenting stress emerged as important proximal pathways associated with postpartum depressive symptoms. Self-perceived burden reflects mothers’ perceptions that their health condition, recovery needs, or caregiving demands negatively affect family members, potentially leading to feelings of guilt, inadequacy, and emotional distress. Previous studies have similarly linked self-perceived burden with depressive symptoms among women experiencing pregnancy-related complications and other chronic health conditions [38,64]. Parenting stress also demonstrated a significant direct association with postpartum depressive symptoms, suggesting that caregiving demands, sleep disruption, role adjustment, and emotional exhaustion may be closely related to depressive symptoms during the postpartum period. This finding is consistent with previous research identifying parenting stress as a key correlate of postpartum depression across diverse maternal populations [65,66]. Together, these findings support the Stress Process Model’s proposition that secondary stressors may represent more immediate pathways through which broader life challenges influence mental health outcomes.

Among the psychosocial resources examined, social support demonstrated the strongest inverse association with postpartum depressive symptoms, showing both direct and indirect inverse associations. This finding suggests that perceived support may not only be associated with lower depressive symptoms directly but may also be associated with reduced psychological distress through lower self-perceived burden and parenting stress. In the Thai context, where postpartum care frequently involves support from partners, family members, and extended kin networks, social support may play an especially important role in facilitating maternal adjustment and reducing caregiving strain. This interpretation is consistent with previous evidence indicating that inadequate social support increases the risk of postpartum depression, whereas family and partner support contribute to improved maternal psychological well-being [44,45].

Mastery demonstrated a significant indirect association with postpartum depressive symptoms, although its direct association was not significant. This finding suggests that higher perceived control may be associated with lower levels of self-perceived burden and parenting stress, which were in turn associated with postpartum depressive symptoms. Mothers with higher levels of mastery may be more likely to perceive postpartum challenges as manageable and therefore experience lower levels of self-perceived burden and parenting stress. This interpretation is consistent with the Stress Process Model, which conceptualizes mastery as a personal resource that may shape responses to stress and modify pathways linking stressors to mental health outcomes [23,24]. However, the mastery construct demonstrated composite reliability and average variance extracted below the recommended thresholds. Therefore, although its indirect association was statistically significant, this finding should be interpreted cautiously because the construct may not have been measured with sufficient reliability and convergent validity.

In contrast, self-esteem and coping did not demonstrate statistically significant direct, indirect, or total associations with postpartum depressive symptoms in the final model. Notably, both self-esteem and coping were significantly and inversely correlated with postpartum depressive symptoms at the bivariate level, but neither retained a significant unique association when all constructs were examined simultaneously. This divergence suggests that their bivariate relationships may partly reflect variance shared with more proximal stressors and psychosocial resources included in the model. In a multivariable structural model, each coefficient represents the association of a construct with postpartum depressive symptoms after accounting for the other variables. Therefore, the nonsignificant findings do not necessarily indicate that self-esteem and coping are unimportant; rather, their unique associations may have been reduced after accounting for self-perceived burden, parenting stress, social support, and mastery.

The nonsignificant association of self-esteem may also reflect conceptual and contextual specificity. The Rosenberg Self-Esteem Scale assesses global self-worth, whereas psychological adjustment during the early postpartum period may be more closely related to maternal role-specific confidence, perceived competence in infant care, role strain, and perceived control. Thus, a measure of global self-esteem may not have captured the aspect of self-evaluation most relevant to mothers during the 4–6-week postpartum period. In addition, maternal psychological well-being may be strongly embedded within interpersonal and family relationships. For example, partner conflict has been associated with postpartum depressive symptoms among Thai mothers [67]. It is therefore possible that relational resources and maternal-role experiences were more salient than global self-worth in the present context. This interpretation remains tentative because maternal self-efficacy, maternal role competence, and relationship quality were not directly examined in the current model.

The nonsignificant association of coping may reflect both conceptual and measurement-related limitations. The coping construct combined problem-focused, active emotional, and avoidant emotional coping strategies, although these strategies may have different or even opposing relationships with postpartum depressive symptoms. Previous studies have suggested that adaptive and maladaptive coping strategies may differentially influence psychological outcomes, and combining multiple coping dimensions into a single latent construct may therefore obscure strategy-specific associations [68,69]. Furthermore, the coping indicators showed heterogeneous standardized factor loadings, and the construct had CR and AVE values below the recommended thresholds. These measurement weaknesses may have attenuated the magnitude and precision of its estimated structural associations. The factor structure of the Brief COPE has also been shown to vary across perinatal stages and populations, with different solutions identified among antenatal and postpartum women [70]. Accordingly, the nonsignificant coping pathways should be interpreted cautiously and should not be considered conclusive evidence that coping is unrelated to postpartum depressive symptoms.

Cultural and contextual factors may also have influenced the measurement and expression of coping. General coping instruments may not fully capture relational, family-based, acceptance-oriented, or culturally meaningful coping responses used by Thai postpartum women. However, because these forms of coping were not specifically assessed, this explanation should be regarded as a hypothesis for further investigation rather than a conclusion from the present findings.

Future longitudinal studies should use postpartum-specific measures of maternal self-efficacy and examine adaptive and maladaptive coping strategies separately. Further psychometric evaluation of coping measures among Thai postpartum women is also needed to determine whether their dimensional structure adequately represents culturally and contextually relevant responses. Such studies could clarify whether self-esteem and coping have delayed, indirect, or context-dependent associations with postpartum depressive symptoms.

Despite these contributions, several limitations should be considered when interpreting the findings. First, the cross-sectional design precludes conclusions regarding the temporal or causal direction of the observed relationships. Accordingly, the identified pathways should be interpreted as theoretically informed associations rather than confirmed causal sequences. Second, participants were recruited from mothers attending routine postpartum follow-up clinics at tertiary hospitals who agreed to participate. Therefore, selection bias cannot be excluded because mothers who did not attend follow-up care or declined participation may have differed in important clinical or psychosocial characteristics. In addition, recruitment from tertiary hospitals may limit the generalizability of the findings to mothers receiving care in community, rural, or primary care settings. Third, the psychosocial variables and postpartum depressive symptoms were assessed at a single time point during the 4–6-week postpartum period and therefore may not fully capture changes in stress processes and depressive symptoms over time. Fourth, the proposed model was intentionally focused and parsimonious rather than comprehensive. Although theory-driven, it did not incorporate several established determinants of postpartum depressive symptoms, including prior or antenatal mental health vulnerability, pregnancy intention, marital or partner relationship quality, infant temperament, and multidimensional socioeconomic adversity. Although women with documented pre-pregnancy depression or psychiatric treatment were excluded, unrecognized or subclinical mental health vulnerability may have remained. In addition, education, occupation, and household income were recorded as sociodemographic characteristics, but these indicators did not constitute a validated multidimensional measure of socioeconomic adversity. The omission of these factors may have introduced residual confounding and limited the explanatory scope of the model. Fifth, the mastery and coping constructs demonstrated composite reliability and average variance extracted values below the recommended thresholds. Although these constructs were retained to preserve the prespecified theoretical model, findings involving mastery, and particularly coping, should be interpreted cautiously. These measurement limitations may have affected the precision and interpretation of the estimated associations involving these constructs and should be considered when generalizing the findings to other postpartum populations. Sixth, the perinatal complications score was calculated as an unweighted cumulative count in which each documented condition contributed equally, without accounting for differences in severity, duration, treatment intensity, or psychological impact. This approach may have underestimated the contribution of severe but infrequent complications or overrepresented that of milder, more common conditions, thereby affecting the estimated associations. Future studies should further evaluate the psychometric properties of mastery and coping measures among Thai postpartum women and examine adaptive and maladaptive coping strategies separately.

## 6. Conclusions

This study tested a Stress Process Model of postpartum depressive symptoms among Thai mothers. The final model demonstrated acceptable fit and explained 65.10% of the variance in postpartum depressive symptoms. Perinatal complications were directly associated with postpartum depressive symptoms and indirectly associated through self-perceived burden and parenting stress. Social support showed the strongest inverse association, while mastery showed an indirect inverse association. The findings support the utility of the Stress Process Model as a framework for organizing factors associated with postpartum depressive symptoms in this population. Assessment of perinatal complications, self-perceived burden, parenting stress, and social support may help identify mothers requiring additional support and inform the development of psychosocial support strategies during the postpartum period. Because all variables were assessed concurrently, the identified pathways should be interpreted as theoretically informed statistical associations rather than evidence of temporal ordering, causal mechanisms, or causal mediation.

## Figures and Tables

**Table 1:** Sociodemographic and Obstetric Characteristics of Participants (*N* = 462).

Characteristics	*N* (%)
**Age** (years) (mean = 30.98, SD = 5.07, range = 20–47)	
20–29	181 (39.18)
30–39	263 (56.93)
≥40	18 (3.89)
**Marital status**	
Married	387 (83.77)
Separated/Divorced/Widowed	75 (16.23)
**Education level**	
High school/Vocational certificate or lower	197 (42.64)
Associate degree/Higher vocational certificate	93 (20.13)
Bachelor’s degree or higher	172 (37.23)
**Occupation**	
Unemployed/Housewife	103 (22.30)
Salaried employee	287 (62.12)
Business owner/Self-employed/Others	72 (15.58)
**Monthly family income in Thai baht (USD)**	
≤10,000 (≤294)	99 (21.43)
10,001–20,000 (295–588)	259 (56.06)
≥20,001 (≥589)	104 (22.51)
**Family structure**	
Nuclear family	190 (41.12)
Extended family	263 (57.93)
Living alone	9 (1.95)
**Gravida**	
Primigravida	211 (45.67)
Multigravida	251 (54.33)
**Number of living children**	
1	241 (52.16)
2	175 (37.88)
≥3	46 (9.96)
**Gestational age at delivery**	
Preterm (<37 weeks)	45 (9.74)
Term (≥37 weeks)	417 (90.26)
**Mode of delivery**	
Vaginal delivery	313 (67.75)
Assisted delivery (VE or FE)	23 (4.98)
Cesarean section	126 (27.27)
**Neonatal complications**	
No	407 (88.09)
Yes (Preterm, Hypoglycemia, Hyperbilirubinemia)	55 (11.91)
**Duration of neonatal hospitalization**	
≤5 days	407 (88.10)
>5 days	55 (11.90)
**Hospital admission during pregnancy**	
No	431 (93.29)
Yes (PIH, Preterm, APH)	31 (6.71)

**Table 2:** Number and Percentage of Participants with postpartum depressive symptoms (n = 462).

Postpartum Depressive Symptoms	*N* (%)
Postpartum depressive symptoms (EPDS score: mean = 7.74, SD = 4.72, Range 0–18)	
Yes (EPDS ≥ 11)	143 (30.95)
No (EPDS < 11)	319 (69.05)

**Table 3:** Standardized Factor Loadings, Composite Reliability, and Average Variance Extracted for the Measurement Model.

Latent Constructs	Indicators	Standardized Factor Loading	CR	AVE	Interpretation
Self-perceived burden	SPB1–SPB3	0.763–0.946	0.913	0.780	Adequate
Parenting stress	PS1–PS4	0.763–0.784	0.857	0.599	Adequate
Social support	SS1–SS3	0.692–0.823	0.819	0.603	Adequate
Self-esteem	SE1–SE3	0.706–0.848	0.820	0.604	Adequate
Mastery	M1–M2	0.556–0.683	0.556	0.388	Below recommended thresholds
Coping	CO1–CO3	0.197–0.751	0.477	0.272	Below recommended thresholds

Note. CR = composite reliability; AVE = average variance extracted. CR values ≥ 0.70 and AVE values ≥ 0.50 were considered indicative of acceptable construct reliability and convergent validity, respectively. Mastery and coping did not meet these recommended thresholds; therefore, findings involving these constructs should be interpreted cautiously.

**Table 4:** Intercorrelations Among Study Variables.

Variables	Com	SPB	PS	SS	SE	M	CO	PDS
Com	1.00							
SPB	0.57 **	1.00						
PS	0.63 **	0.66 **	1.00					
SS	−0.57 **	−0.45 **	−0.54 **	1.00				
SE	−0.34 **	−0.30 **	−0.36 **	0.30 **	1.00			
M	−0.42 **	−0.38 **	−0.45 **	0.40 **	0.49 **	1.00		
CO	−0.27 **	−0.33 **	−0.37 **	0.31 **	0.29 **	0.28 **	1.00	
PDS	0.67 **	0.62 **	0.66 **	−0.61 **	−0.42 **	−0.46 **	−0.28 **	1.00

Note. Variables; Com = perinatal complications, SPB = self-perceived burden, PS = parenting stress, SS = social support, SE = self-esteem, M = mastery, CO = coping, PDS = postpartum depressive symptoms (EPDS total score). ** *p* < 0.01.

**Table 5:** Sequential Respecification and Fit Indices of the Structural Model.

Step	User-Specified Respecification	MI	StdYX EPC	χ^2^	df	χ^2^/df	RMSEA	SRMR	CFI	TLI
Initial model	None	—	—	592.368	149	3.98	0.080	0.085	0.904	0.877
Step 1	CO3 WITH CO2	20.779	0.244	570.812	148	3.86	0.079	0.085	0.908	0.880
Step 2	PS4 WITH PS3	27.030	−0.322	537.387	147	3.66	0.076	0.085	0.915	0.890
Step 3	PS2 WITH PS1	26.567	−0.303	507.707	146	3.48	0.073	0.085	0.921	0.898
Step 4	C2 WITH SS †	24.419	−0.215	430.608	143	3.01	0.066	0.047	0.938	0.917
Final model	PS WITH SPB	10.656	6.016	419.363	142	2.95	0.065	0.046	0.940	0.919

Note. MI = modification index; StdYX EPC = fully standardized expected parameter change; RMSEA = root mean square error of approximation; SRMR = standardized root mean square residual; CFI = comparative fit index; TLI = Tucker–Lewis index. MI and StdYX EPC values were obtained from the immediately preceding model. Models were re-estimated after each respecification. † At Step 4, C2 WITH SS incorporated the observed perinatal complications variable into the modeled covariance structure, producing additional C2-related parameter estimation and a three-degree-of-freedom reduction. No structural regression (ON) or measurement (BY) specifications were changed.

**Table 6:** Standardized Direct, Indirect, and Total Associations with Postpartum Depressive Symptoms.

Predictor	Direct Association (β)	Indirect Association (β)	Total Association (β)
Perinatal Complications	0.17 ***	0.15 **	0.32 ***
Self-Perceived Burden	0.22 ***	-	0.22 ***
Parenting Stress	0.18 **	-	0.18 **
Social Support	−0.30 ***	−0.11 **	−0.41 ***
Mastery	−0.11	−0.13 *	−0.24 *
Self-Esteem	−0.09	0.03	−0.06
Coping	−0.04	−0.05	−0.09
R^2^			0.65

Note. β = standardized coefficient. Direct, indirect, and total associations represent statistical estimates within the structural equation model and should not be interpreted as causal effects. * *p* < 0.05, ** *p* < 0.01, *** *p* < 0.001.

## Data Availability

The data presented in this study are available from the corresponding author upon reasonable request. The data are not publicly available due to ethical and privacy restrictions.

## Public Involvement Statement

No patients or members of the public were involved in the design, conduct, reporting, or dissemination plans of this research.

## Guidelines and Standards Statement

This manuscript was prepared in accordance with the Strengthening the Reporting of Observational Studies in Epidemiology (STROBE) Statement for reporting observational studies.
